# The spike-and-slab elastic net as a classification tool in Alzheimer’s disease

**DOI:** 10.1371/journal.pone.0262367

**Published:** 2022-02-03

**Authors:** Justin M. Leach, Lloyd J. Edwards, Rajesh Kana, Kristina Visscher, Nengjun Yi, Inmaculada Aban, for the Alzheimer’s Disease Neuroimaging Initiative

**Affiliations:** 1 Department of Biostatistics, University of Alabama at Birmingham, Birmingham, Alabama, United States of America; 2 Department of Psychology, University of Alabama, Tuscaloosa, Alabama, United States of America; 3 Department of Neurobiology, University of Alabama at Birmingham, Birmingham, Alabama, United States of America; Icahn School of Medicine at Mount Sinai, UNITED STATES

## Abstract

Alzheimer’s disease (AD) is the leading cause of dementia and has received considerable research attention, including using neuroimaging biomarkers to classify patients and/or predict disease progression. Generalized linear models, e.g., logistic regression, can be used as classifiers, but since the spatial measurements are correlated and often outnumber subjects, penalized and/or Bayesian models will be identifiable, while classical models often will not. Many useful models, e.g., the elastic net and spike-and-slab lasso, perform automatic variable selection, which removes extraneous predictors and reduces model variance, but neither model exploits spatial information in selecting variables. Spatial information can be incorporated into variable selection by placing intrinsic autoregressive priors on the logit probabilities of inclusion within a spike-and-slab elastic net framework. We demonstrate the ability of this framework to improve classification performance by using cortical thickness and tau-PET images from the Alzheimer’s Disease Neuroimaging Initiative (ADNI) to classify subjects as cognitively normal or having dementia, and by using a simulation study to examine model performance using finer resolution images.

## Introduction

### Alzheimer’s disease overview

Dementia has a long history as a scourge on the quality of life for aging persons, and in recent decades has been a leading cause of death [1, 2]. The medical research community has thus devoted considerable time and effort to understanding dementia’s leading cause, Alzheimer’s disease (AD). In consequence, the understanding of AD, and dementia in general, has developed significantly in the last century, and is evolving still [1].

Biomarker research has progressed alongside an increased understanding of AD’s etiology. AD pathology is characterized primarily by amyloid plaques and neurofibrillary tangles [2]. The amyloid cascade hypothesis proposes that amyloid deposits lead to abnormal tau protein aggregation, which then lead to the neurofibrillary tangles that damage neurons and result in deficits in cognitive and functional ability [3, 4]. However, subsequent research casts significant doubt on the viability of this hypothesis, specifically related to its temporal assumptions; while alternative theories have arisen, a stable consensus about these theories is yet to be achieved [5–7]. Given this uncertainty, temporal agnosticism is the current norm with respect to biomarker-related risks [1, 8].

The popularity of the amyloid cascade hypothesis drove significant focus on amyloid-pathology-related imaging approaches, but doubt surrounding the hypothesis’ validity, and evidence that tau-pathology more closely aligns with disease severity, created an interest in imaging approaches that capture tau protein pathology [9]. To date, Positron Emisson Tomography (PET) imaging with the tracer [^18^F]AV-1451, or flortaucipir, is the most widely used image modality for tau protein imaging. Another important biomarker, brain atrophy, predates the amyloid cascade hypothesis, indicates the extent of neurodegeneration, and is correlated with both tau deposition and neuropsychological deficits [10]. Brain atrophy is not confined to AD, and should generally not be used in isolation to diagnose AD, but nevertheless patterns of brain atrophy have been useful in AD research [10].

### Classification and model selection

**Fig 1:**
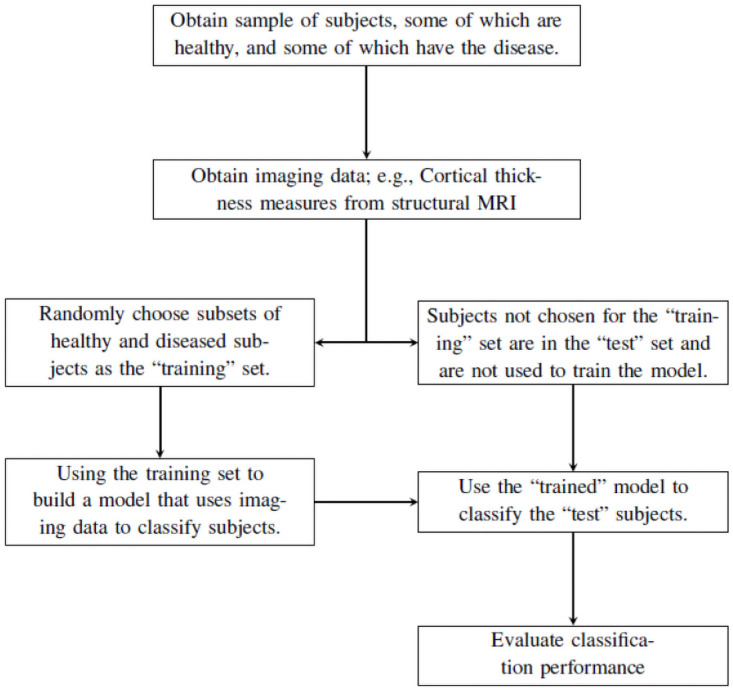
This flow chart describes the basic process of building and evaluating a classifier.

The availability of neuroimaging-based biomarkers resulted in research on algorithmic approaches to classification of, and prediction of progression to, MCI and AD [1, 11, 12]. Often the task is binary classification, i.e., imaging data is input to the algorithm/model, which classifies the subject as having or not having the disease. For a given classification paradigm, we must select the “optimal” model and provide an objective description of how well the model distinguishes between subjects with or without the disease. These are related issues since the criteria for the optimal model/classifier is how well the classifier classifies subjects it has not yet seen. The general process, shown in Fig 1, divides data into “training” and “test” sets. Subjects in the training set are used to fit the model, which is used to predict the classes for subjects in the test set, after which we evaluate the classifier’s performance.

Many classifiers have tuning parameters that must be chosen by the investigator, and in Bayesian contexts, tuning parameters usually correspond to the hyperparameters in prior distributions. Classification problems require wariness towards overfitting models, because to be useful a model must generalize to new data. Thus, tuning parameter value choices should be based on corresponding estimates of expected prediction error. Prediction error estimates can be obtained via k-fold cross validation [13]. K-fold cross validation divides the subjects into *k* independent sets, and fits the model *k* times, each time holding out a different set as the test set. The average prediction error across the sets provides an estimate for the expected prediction error, and parameter values that minimize the estimate of expected prediction error are used to fit the classifier/model using all available data. Since many datasets have too small a sample size to hold out a separate test set, and since k-fold cross validation provides estimates of expected prediction error, it also allows for evaluating model performance while making use of all available data to build the model.

Improvement in the accuracy of these algorithms has the potential to allow for earlier detection of MCI and/or AD, and if sufficiently accurate in predicting progression, may allow researchers to identify patients who are at high risk for progression to AD. While many algorithms used thus far have shown promise, statistical learning approaches continue to advance, and it is beneficial to apply, extend, and evaluate these algorithms with respect to MCI/AD classification. In this work we add to the classification literature by applying and evaluating the performance of the spike-and-slab elastic net with spatially informative priors as a classifier. First, we review several relevant statistical methods that build up to the methods used in this paper.

### Penalized and Bayesian generalized linear models

Penalized generalized linear models are a useful approach to statistical learning, the most well-known of which are the ridge, lasso, and elastic net; the latter compromises between the first two [14, 15]. All three of these models have Bayesian interpretations, and the latter two are useful for variable selection, because they tend to produce sparse solutions, i.e., applying the penalty results in many, if not most, of the parameter estimates being zero which allows for variable selection without resorting to null hypothesis significance testing (NHST). While there are some exceptions, these models generally produce estimates under ill-posed data, i.e., when predictors outnumber subjects or observations [15].

The elastic net avoids pitfalls of both the ridge and lasso. The ridge cannot provide automatic variable selection because all parameter estimates are non-zero, and when the number of predictors far outnumbers the number of subjects, the number of non-zero estimates allowed from the lasso is capped at the number of subjects. A related issue is when predictors are highly correlated, the lasso tends to choose one and discard the rest. In contrast, the elastic net provides sparse solutions while leaving more parameter estimates non-zero, making it an attractive approach when using images as predictors, since predictors often outnumber subjects, and “relevant” predictors may spatially cluster and be highly correlated. While penalty parameters, or prior distributions, must be chosen by the researcher, cross validation provides a principled approach to penalty parameter selection.

While penalized models produce biased estimates, the trade-off is typically substantial reduction in variance around the parameter estimates and predicted outcomes, which can improve the generalizeability of the models. The elastic net framework is thus attractive for both variable selection and prediction, and can be adapted to classification problems, e.g., by applying classification rules to a penalized/Bayesian logistic regression. An interesting consequence of the elastic net is that the sparse solutions produced by the initial model mean that the classifier will remove “unnecessary” predictors/features during the model fitting process by shrinking their estimates to zero, so that only a subset of the initial set of possible predictors/features is used in the classifier; the automatic variable selection of elastic net models equates to automatic feature selection in a statistical learning context.

The Bayesian interpretation of these models allows for useful extensions with respect to variable selection. In particular, the spike-and-slab lasso combines the lasso penalty with a common Bayesian approach to variable selection: the spike-and-slab prior, which models parameters as arising from a mixture of two distributions, one each for parameters that are and are not relevant to modeling the outcome of interest, respectively [16–19]. This approach extends the lasso such that there is stronger shrinkage imposed on parameters that are irrelevant and weaker shrinkage applied to relevant parameters, which allows the final estimates of relevant parameters to remain relatively large, and drives estimates of irrelevant parameters to zero.

### Outline

The elastic net is applicable to a wide range of problems, including imaging data, especially since it can perform variable/feature selection when predictors are correlated and/or outnumber subjects. The elastic net has been used in other AD classification studies [20–23]; however, the combination of spike-and-slab priors with the elastic net framework is a relatively new methodology, and to our knowledge it has not yet been explored in AD classification, or medical imaging more generally. The primary aim of this work is to demonstrate the utility of this methodology as a classifier using medical images in general, and within AD in particular.

In previous work, we extended the spike-and-slab lasso to accommodate the elastic net penalty and explicitly model dependence among predictors, and showed that both extensions contributed to improved model performance [24]. This class of models contains (Bayesian) logistic regression as a special case, and by using thresholding rules we can create a classifier. That is, the parameter estimates from a logistic regression can produce estimated/predicted probabilities of subjects having the disease. Thresholds are applied to the probabilities to classify subjects; e.g., we can classify subjects with predicted probabilities of disease above 0.5 as having the disease.

In what follows, we demonstrate the classification utility of the spike-and-slab elastic net with spatially structured priors by using the model to classify subjects as being cognitively normal or having dementia using data from the Alzheimer’s Disease Neuroimaging Initiative (ADNI). The outline is as follows: we briefly describe the ADNI data, discuss specific methods and outcomes used for classification, review the statistical details relevant to understanding the classifiers, and discuss several metrics used to evaluate classification performance. We then use ADNI data to classify subjects’ disease status using cortical thickness and tau PET images as predictors, and compare the results across several classifiers built from the methods presented in the preceding section. We also present a simulation study to examine the model performance using higher resolution images. Finally, we discuss the implications of the results, outline future research directions, and address limitations in the present study.

## Materials and methods

### ADNI details

Data used in the preparation of this article were obtained from the Alzheimer’s Disease Neuroimaging Initiative (ADNI) database (adni.loni.usc.edu). The ADNI was launched in 2003 as a public-private partnership, led by Principal Investigator Michael W. Weiner, MD. The primary goal of ADNI has been to test whether serial magnetic resonance imaging (MRI), positron emission tomography (PET), other biological markers, and clinical and neuropsychological assessment can be combined to measure the progression of mild cognitive impairment (MCI) and early Alzheimer’s disease (AD).

As mentioned in the introduction, brain atrophy is a useful metric for studying AD. A related measure is cortical thickness, which can be assessed in a cross sectional setting, whereas true atrophy measures would require longitudinal measures. Cortical thickness measures have been used as classification features throughout the literature [12]. However, cortical thickness is not trivial to measure because the topology of the cortex is that of a densely folded 2D sheet, and manual measurements are laborious and error-prone. In contrast, computational approaches to “unfolding” the cortex can result in accurate assessments of cortical properties, including cortical thickness. One such approach starts by segmenting white and grey matter voxels, then estimates the white/grey boundary at the subvoxel level using a triangular tessellation, and finally “inflates” that boundary out towards the pial surface to obtain the outer boundary, minimizing metric distortions along the way [25, 26]. Accurate cortical thickness measures are obtained by finding the distance between these two surfaces at a given point [27]. FreeSurfer software performs this whole procedure, resulting in a wide array of data, including surface area, volume, and cortical thickness measures [28]. FreeSurfer-processed ADNI data is available as summaries for brain regions specified by Desikan-Killiany atlas [29].

In addition to MRI, ADNI-3, the most recent renewal of the ongoing ADNI study, also has [^18^F]AV-1451 Positron Emisson Tomography (PET) imaging. In fact, one of the motivations of ADNI-3 was to incorporate “innovative technologies”, which includes a focus on tau PET imaging [30]. In ADNI tau PET images have been processed with Freesurfer, and standardized uptake value ratios (SUVR) summaries are available by regions of the Desikan-Killiany atlas.

Since both cortical thickness and tau PET images can be reasonably expected to provide information regarding AD status, in the present work we pursue two separate paths of analysis: one using cortical thickness summaries as features, and the other using tau PET SUVR summaries as features.

### Statistical methods

Logistic regression arises from a generalized linear model (GLM) where outcomes are binary; subject-specific probabilities of being in one class or the other can be extracted from such models, and we can then apply thresholding rules to build a classifier. The mathematical form of a GLM is as follows:

$$\begin{array}{cc} g(\text{E}\left(y_{i}|X_{i}\right)) & =X_{i}\beta =\beta_{0}+\sum_{j=1}^{J}x_{ij}\beta_{j}=\eta_{i},\;i=1,\ldots ,N \end{array}$$ (1)

where for the *i*^*th*^ subject, *y*_*i*_ is an observed outcome and the *y*_*i*_’s are independent, ***X***_*i*_ is 1 × (*J* + 1) vector of measured predictors, ***β*** is a (*J* + 1) × 1 vector of unknown parameters, and *g*(⋅) is an appropriate link function. In this work, the outcome is disease class, e.g., whether the subject is cognitive normal (CN) or has dementia. We thus assume the outcome has a binomial distribution and use logistic regression, so that *g*(⋅) is the logit function. The predictors are the subject’s average cortical thickness or tau disposition measures for each region in Desikan-Killiany atlas, which divides each hemisphere of the cerebral cortex into 34 regions. Thus, each subject will have a single outcome indicating disease status, and sixty-eight predictors (*J* = 68), one for each region of the Desikan-Killiany atlas.

Spike-and-slab priors are a mixture of distributions. The wider distribution (slab) has large variance to impose weak penalties and the narrower distribution (spike) has small variance to impose strong penalties:

$$\begin{array}{cc} p(\beta_{j}|\gamma_{j}) & =\gamma_{j}p_{1}\left(\beta_{j}|\gamma_{j}=1\right)+\left(1-\gamma_{j}\right)p_{0}\left(\beta_{j}|\gamma_{j}=0\right) \end{array}$$ (2)

where *γ*_*j*_ is a binary indicator variable of model inclusion [16, 17]; i.e., *γ*_*j*_ = 1 indicates the *j*^*th*^ predictor should remain in the model and *γ*_*j*_ = 0 indicates it should not. The spike-and-slab lasso combines spike-and-slab priors with the lasso penalty, which in Bayesian terms correspond to double exponential distributions for *p*_1_ and *p*_0_ in Eq (2), and penalizes estimates for “irrelevant” parameters more strongly than those of “relevant” parameters, which shrinks more “irrelevant” parameter estimates to zero, while allowing “relevant” parameter estimates to remain larger [19]. The original framework focused on linear models, but can be extended and applied in other contexts; e.g., spike-and-slab lasso GLM’s are fit by using an Expectation-Maximization Coordinate Descent algorithm [31, 32]. While there are other shrinkage priors in the literature, most notably the horseshoe prior [33], we focus on the spike-and-slab lasso and its extensions rather than the horseshoe prior. A primary reason is that there exist comparatively fast algorithms for fitting spike-and-slab lasso models, making them more attractive for high-dimensional correlated data [19, 31]. Additionally, our focus is on incorporating spatial information into these algorithms, which is both more naturally implemented in the spike-and-slab framework, and given that adding such information necessarily adds computational time it makes sense to focus on models for which model fitting is already relatively quick [24].

As discussed above, the lasso penalty may have downsides when predictors arise from images, and as we show in prior work, the spike-and-slab lasso can be generalized to a spike-and-slab elastic net prior [24]:

$$\begin{array}{cc} p(\beta_{j}|\gamma_{j},s_{0},s_{1}) & =EN(\beta_{j}|0,S_{j})\\ & =\left(1-\xi \right)\text{exp}(-\text{log}\left(\sqrt{2\pi S_{j}}\right)-\frac{\beta_{j}^{2}}{S_{j}})\\ & \;+\xi \text{exp}(-\text{log}\left(2S_{j}\right)-\frac{|\beta_{j}|}{S_{j}}) \end{array}$$ (3)

where *S*_*j*_ = (1 − *γ*_*j*_)*s*_0_ + *γ*_*j*_*s*_1_, *s*_1_ is the slab scale, *s*_0_ is the spike scale, *s*_1_ > *s*_0_ > 0, and *ξ* ∈ [0, 1]. The wider “slab” distribution allows parameters to take larger values, while the narrower “spike” distribution shrinks estimates severely toward zero. A spike-and-slab ridge is obtained when *ξ* = 0 and spike-and-slab lasso when *ξ* = 1. The *γ*_*j*_ ∈ {0, 1} are indicator variables for model inclusion, and are assigned a Bernoulli distribution with unknown probability of inclusion given by *θ*_*j*_.

Since we do not know a priori which predictors should remain in the model, it is necessary to assign prior distribution(s) for the indicator variables, *γ*_*j*_. In the simplest setting, we may assume that the *γ*_*j*_ have a Bernoulli prior with global or predictor-specific probability of inclusion *θ* or *θ*_*j*_, respectively:

$$\begin{array}{cc} p(\gamma_{j}|\theta_{j}) & =\theta_{j}^{\gamma_{j}}{\left(1-\theta_{j}\right)}^{1-\gamma_{j}} \end{array}$$ (4)

However, in spatial applications spatial information is often relevant to determining which parameters affect an outcome; i.e., we expect relevant parameters to cluster spatially. For example, many multiple testing procedures in neuroimaging explicitly make this assumption by taking into account the size, extent, and general properties of clusters of statistically significant voxels [34]. Unlike classical GLM’s, Bayesian GLM’s will provide solutions when predictors are highly correlated, but will not incorporate spatial information into model selection unless explicitly included in the prior distributions. It is conceivable to explicitly model correlation among the parameters, *β*_*j*_, in their prior distributions, but a more computationally viable approach is to model correlation among the prior probabilities of inclusion, *θ*_*j*_, whose conditional estimates affect the degree of shrinkage applied to parameters. Correlation among these estimates mean that a given estimate for *β*_*j*_ depends in part upon the shrinkage applied to its neighbors’ estimates. A variant of Conditional Autoregressions (CAR) known as Intrinsic Autoregressions (IAR) have been used to incorporate spatial information into a wide range of practical applications, and can be used as a prior distribution on the logit probabilities of inclusion [35–38]. Below is the prior distribution for the logit of probabilities of inclusion:

$$\begin{array}{cc} \text{log}\;p(\psi_{j}|\psi_{i},\tau ) & \propto \frac{-\tau^{2}}{2}(\sum_{j∼i}{\left(\psi_{j}-\psi_{i}\right)}^{2}) \end{array}$$ (5)

where *ψ*_*j*_ = logit(*θ*_*j*_) and *j* ∼ *i* indicates the location/predictor *i* is a neighbor of location/predictor *j*, i.e., the summation is over all the neighbors of location/predictor *j*. Note that in practice, we often set *τ* = 1, which is the convention applied in this work [39]. The information from Eqs (1), (3), (4), and (5) combine to form the following log joint posterior:

$$\begin{array}{cc} \text{log}\;p(\beta ,\phi ,\gamma ,\psi |y) & \propto \underset{\text{log}\;\text{likelihood}}{\underset{︸}{\ell (\beta ,\phi )}}-\underset{\text{log}\;\text{prior}\;\text{for}\;\beta }{\underset{︸}{\sum_{j=1}^{J}\text{log}\;EN\left(\beta_{j}|0,S_{j}\right)}}\\ & \;+\underset{\text{log}\;\text{prior}\;\text{for}\;\gamma }{\underset{︸}{\sum_{j=1}^{J}\gamma_{j}\text{log}\;\theta_{j}+\left(1-\gamma_{j}\right)\text{log}\left(1-\theta_{j}\right)}}\\ & \;-\underset{\text{log}\;\text{prior}\;\text{for}\;\psi_{j}=\text{logit}\left(\theta_{j}\right)}{\underset{︸}{\frac{1}{2}(\sum_{j∼i}{\left(\psi_{j}-\psi_{i}\right)}^{2})}} \end{array}$$ (6)

The model described by Eq (6) can be fit with an Expectation-Maximization Coordinate Descent algorithm described in prior work [24]. This framework for incorporating spatial structure into variable selection can be computationally preferable to, e.g., imposing Ising or Markov random field priors on the indicators, *γ*_*j*_, because imposing spatial structure on the (logit) inclusion probabilities allows for the E-step to remain essentially unchanged from what it would be if there were no spatial structure. This is because, in effect, the IAR prior can be viewed as spatially smoothing the inclusion probabilities, which is handled in the M-step; complete details of the EM algorithm are described elsewhere [24]. Code for fitting the models is possible using the R package ssnet, which we developed and is freely available on GitHub (https://github.com/jmleach-bst/ssnet).

### Classification and model evaluation

**Fig 2:**
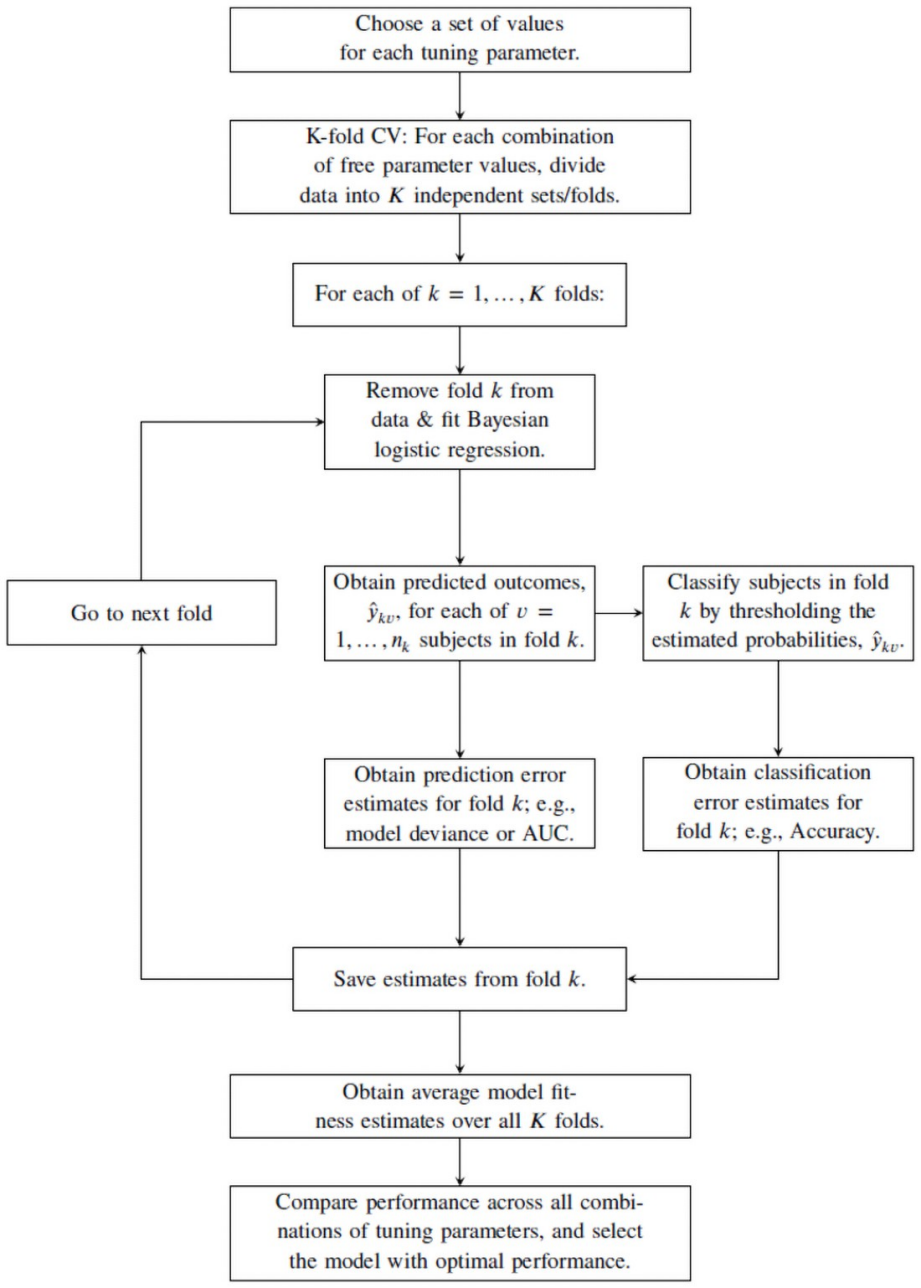
This flowchart describes application of k-fold cross validation employed in this paper.

This work aims to examine the classification ability of spike-and-slab elastic net models. However, the user must select values for several “free” parameters. K-fold cross validation techniques provide a principled way to make such choices, by providing reasonable estimates of prediction error, as assessed by cross-validated estimates of metrics like model deviance, mean-squared error, or area under the ROC curve (AUC) [13]. We fit the model for several values of each free parameter and compare their prediction error estimates, and then select the model with the lowest prediction error estimates as the “best” model. Fig 2 details the implementation of k-fold cross validation used in this work.

The predicted outcomes obtained using parameter estimates from logistic regression are probabilities, which we may be thresholded to classify subjects as either having or not having the disease. In theory any value between zero and one can be used as a threshold, but in practice subjects are typically placed in the class for which they have the highest predicted probability, e.g., subjects with probability of dementia greater than 0.5 are classified as having dementia, and all others classified a cognitively normal. Note that in this application, the assessment of model performance is not with respect to the properties of effect estimates, e.g, odds ratios, but whether the post-threshold classification is accurate when applied to current subjects as well as subjects the model has yet to see.

Assessing classifiers requires evaluation with respect to several metrics in order to avoid being deceived. An obvious concern is the classifier’s accuracy, that is, what is the probability that the algorithm correctly classifies a subject. However, this metric can be misleading, especially in situations where the sample sizes for each class are unbalanced. Thus, in addition to accuracy, it is important to estimate and consider several other metrics in evaluating classification performance:

1. *Sensitivity*: the probability that the subject is classified as having the disease, given that the subject has the disease.
2. *Specificity*: the probability that the subject is not classified as having the disease, given that the subject does not have the disease.
3. *Positive Predictive Value (PPV)*: the probability that a subject has the disease, given that the subject was classified as having the disease.
4. *Negative Predictive Value (NPV)*: the probability that the subject does not have the disease, given that the subject is classified as not having the disease.

In the authors’ experience, PPV and NPV are often neglected, but neglecting these metrics makes the same mistake as only reporting accuracy, because these metrics can also be deceiving, especially under unbalanced data. Unlike sensitivity and specificity, these two metrics depend on the prevalence of the outcome, but nevertheless can provide useful information. Ideally, all five metrics should be near one if the classifier is performing well.

It may also be desirable to evaluate classification performance with measures that evaluate all four confusion matrix (true positives, false positives, true negatives, false negatives). The Matthew’s correlation coefficient (MCC) has advantages over accuracy as well another common summary measure, the F1 score; the form of these metrics is as follows:

$$\begin{array}{cc} \text{MCC} & =\frac{\text{TP}\cdot \text{TN}-\text{FP}\cdot \text{FN}}{\sqrt{\left(\text{TP}+\text{FP})\cdot (\text{TP}+\text{FN})\cdot (\text{TN}+\text{FP})\cdot (\text{TN}+\text{FN}\right)}} \end{array}$$ (7)

$$\begin{array}{cc} \text{F1} & =\frac{2\cdot \text{TP}}{2\cdot \text{TP}+\text{FP}+\text{FN}} \end{array}$$ (8)

where TP is the number of true positives, TN is the number of true negatives, FP is the number of false positives, and FN is the number of false negatives. All metrics described above should be estimated within a cross-validation process because we are most concerned with estimating the metrics’ values when applying the classifier to independent data. Therefore, it is important to bear in mind that we will be assessing the models’ performance on two levels: the first is in basic model selection, that is, regardless of the classification rule, how well do we predict the initial model would generalize. Second, after applying the classification rule to create a classifier, how well do we predict the classifier would classify new subjects? These two levels are the heart of the present examination, with a particular focus on the latter, classification ability.

### ADNI data analysis and simulation study details

#### General analysis approach

We explore the spike-and-slab elastic net as a classifier with an application to data from the ADNI study, after which we further probe classification performance with a simulation study. In both cases we fit two sets of models. The first set of models is fit with the traditional lasso (*ξ* = 1), spike-and-slab lasso (SSL), and spike-and-slab lasso with IAR priors on the inclusion probabilities (SSL-IAR). The second set of models is a halfway compromise between the ridge and lasso (*ξ* = 0.5), resulting in what we refer to as the traditional elastic net (EN), spike-and-slab elastic net (SSEN), and spike-and-slab elastic net with IAR priors on the inclusion priors (SSEN-IAR). Note that the traditional elastic net can be obtained by maximizing only the first line of Eq (6), i.e., $\ell \left(\beta ,\phi \right)-\sum_{j=1}^{J}\text{log}\;EN\left(\beta_{j}|0,S_{j}\right)$, and by simplifying the form of *S*_*j*_ from *S*_*j*_ = (1 − *γ*_*j*_)*s*_0_ + *γ*_*j*_*s*_1_ to a single penalty *S*_*j*_ = *λ*. Recall that including IAR priors on inclusion probabilities is how we explicitly model spatial structure; thus, the three levels of models can be seen as gradually extending the elastic net from its traditional form to spike-and-slab form to a spike-and-slab form with an explicit modeling of spatial structure. For a given model and set values of *s*_1_ and *s*_0_ we obtain model fits and prediction error statistics via 5-fold cross validation.

Prior scale values, i.e., *s*_1_ and *s*_0_, must be selected in a principled way. The traditional models have that *s*_1_ = *s*_0_, in which case a single parameter value must be chosen. We use the R package glmnet to fit a grid of models and select the model whose scale parameter minimizes the cross-validated deviance. When using spike-and-slab priors we use the R package ssnet to fit models on a grid of spike priors, whose choice is discussed in subsequent sections, and select the model that minimizes the cross-validated deviance. That is, we perform 5-fold cross validation to obtain parameter and prediction error estimates at each combination of values for *s*_0_ and *s*_1_, and then choose the values of *s*_0_ and *s*_1_ that minimize the prediction error as measured by cross-validated deviance. While models are selected using deviance, we also report cross-validated estimates of mean squared error (MSE), mean absolute error (MAE), area under the ROC curve (AUC), and misclassification (MC) to enable comparison across models. Note that misclassification is defined as $\frac{1}{N}\sum_{i=1}^{N}I(|y_{i}-{\hat{y}}_{i}\left|>0.5\right)$ where *I*(⋅) is an indicator function whose value is 1 when the argument is true, and zero otherwise.

Classification is performed in each case by placing an observation in the class that has the highest probability; e.g., when comparing CN and dementia, if the estimated probability is >0.5, then the subject is classified as dementia. Classification performance estimates are obtained within the cross validation process. We evaluate the classification performances of each model using estimates for accuracy (AC), sensitivity (SN), specificity (SP), positive predictive value (PPV), negative predictive value (NPV), Matthews Correlation Coefficient (MCC), and F1 score.

#### ADNI data analysis

Two cross sectional datasets from ADNI were employed for classification, one containing cortical thickness measures and one containing standardized uptake value ratios (SUVR) from tau PET images [40]. The cortical thickness data set included 273 subjects, of which 234 (85.71%) were cognitively normal (CN) and 39 (14.29%) had dementia. The tau PET dataset included 303 subjects, of which 262 (86.47%) were cognitively normal (CN) and 41 (13.53%) had dementia. For each data set, we evaluate the algorithm’s ability to classify CN vs. dementia using cortical thickness or tau PET images as predictors. Each data set has 68 predictors, i.e., one predictor for every region of the Desikan-Killiany atlas. For a given model and set values of *s*_1_ and *s*_0_ we obtain model fits and prediction error statistics via 5-fold cross validation; since the resulting held-out sets were relatively small, we performed 5-fold cross validation 10 times each case in order to obtain more stable estimates.

For traditional lasso and elastic net we use the R package glmnet to select the penalty parameter the minimizes cross-validated deviance, and for the spike-and-slab models use the R package ssnet to fit models on a grid of spike priors, *s*_0_ = {0.01, 0.02, …, 0.5}, and slab priors, *s*_1_ = {1, 2, 3, 4, …, 10} and select the model that minimizes the cross-validated deviance.

#### Simulation study details

A limitation of the Desikan-Killiany atlas is that it is a relatively coarse atlas, and the methodology described above can accommodate image resolutions much finer than 68 predictors. The benefit of incorporating spatial information into the models may be more obvious when using finer resolution images. In order to demonstrate and assess model performance at higher resolutions, we performed a simulation study to examine classification performance in a simulation study with similar imbalance in outcome, i.e., large percentages of subjects not having the event of interest, but at a much finer resolution. In contrast to the ADNI data, which consists of 68 predictors, we simulate data with 1,600 predictors. Using the R package sim2Dpredictr (https://github.com/jmleach-bst/sim2Dpredictr) we generated 2,500 simulated data sets with sample size *N* = 250. The dimension of the simulated predictor images was 40 × 40, resulting in 1600 parameters/predictors. The 1600 × 1 parameter vector, ***β***, was generated in 40 × 40 two-dimensional space before vectorization, and a circular region on 49 (3.06%) locations were selected to have non-zero values. We examined two levels of non-zero parameter values, *β*_*j*_ = 0.10 or *β*_*j*_ = 0.05 if location *j* is in the specified circular region and *β*_*j*_ = 0 otherwise (*j* = 1, …, 1600). Subject images were generated on a 40 × 40 two-dimensional lattice using a multivariate Normal distribution with means −1 or −1.25 for parameter sizes 0.10 and 0.05 respectively, unit variance, and a correlation structure where for any two locations on the 2D lattice the correlation between their values was equal to 0.90^*d*^, where *d* is the Euclidean distance between the locations. The images were then vectorized into 1 × 1600 design vectors, ***X***_*i*_, for each of the *i* = 1, …, 250 subjects. Subject outcomes, *y*_*i*_, were generated using a binomial distribution with mean (1 + 1/exp(***X***_*i*_ ***β***))^−1^. Note that the non-zero means used to generate the ***X***_*i*_ were chosen to approximate the imbalance in outcomes seen in the ADNI data, which is described above. The average percentage of “events” of interest for scenarios where non-zero *β*_*j*_ = 0.10 and *β*_*j*_ = 0.05 was 13.84% (min: 7.20%, max: 21.60%) and 13.04% (min: 6.80%, max: 18.40%), respectively. Further details regarding the simulation study and R code for reproducing results can be found at https://github.com/jmleach-bst/ssen-classification-code.

Estimates of the prediction error and classification performance for a given model were obtained using 5-fold cross validation. Optimal values for *s*_0_ and *s*_1_ were chosen from a grid of possible values (*s*_0_ = {0.1, 0.2, 0.3, …, 0.1} and *s*_1_ = {1, 2}) using cross-validated values of deviance.

## Results

### ADNI data analysis

**Fig 3:**
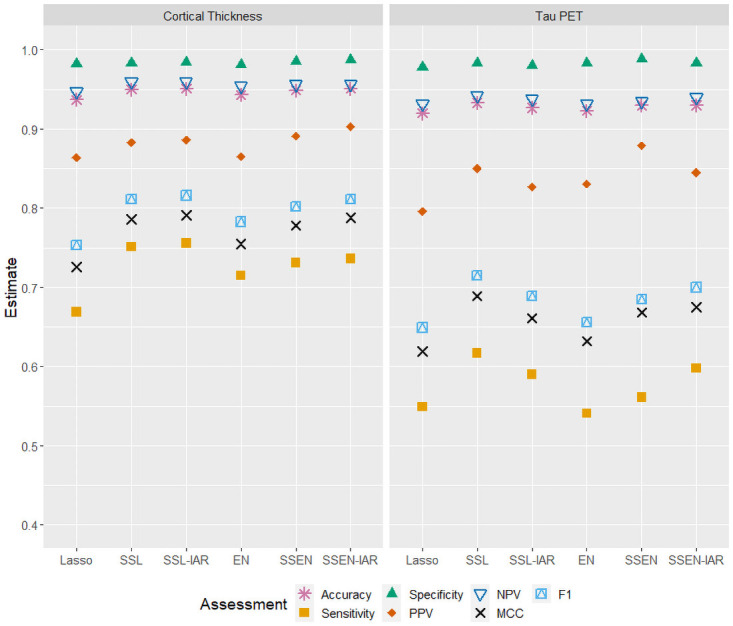
ADNI data classification performance when using cortical thickness measures (LEFT) or tau PET SURV (RIGHT) as predictors/features.

Prediction error estimates for both cortical thickness and tau PET data are displayed in Table 1; model fitness for cortical thickness was explored in other work, but this work did not explore classification performance or utilize tau PET images, and we reproduce model fitness estimates for cortical thickness here for completeness and due to their relevance to the current work [24]. For the cortical thickness data SSEN-IAR has the lowest deviance and MSE, and highest AUC, while SSL has the lowest MAE and misclassification. For the tau PET data, SSL is best performing model for all five prediction error metric estimates. Classification performance metrics are displayed in Table 2 and Fig 3, where we see that accuracy is high for all models in both data sets. SSEN-IAR and SSL-IAR are tied for the highest accuracy, while SSEN-IAR has the highest specificity and PPV, and SSL-IAR has the highest MCC and F1. SSL and SSL-IAR are tied for the highest NPV. In the tau PET data SSL had the best performance with respect to accuracy, sensitivity, NPV, MCC, and F1, while SSEN had the best performance with respect to specificity and PPV.

**Table 1:** ADNI: Prediction error estimates.

	Model	*s*_0_	*s*_1_	Cross-Validated Average				
				Dev.	AUC	MSE	MAE	MC
Cortical Thickness	Lasso	0.002	0.002	90.32	0.952	0.046	0.094	0.063
	SSL	0.270	7.500	73.59	0.969	0.035	**0.067**	**0.050**
	SSL-IAR	0.260	6.000	70.87	0.972	0.035	0.069	0.049
	EN	0.001	0.001	84.26	0.958	0.043	0.088	0.057
	SSEN	0.260	10.000	71.96	0.970	0.036	0.077	0.051
	SSEN-IAR	0.280	10.000	**67.02**	**0.975**	**0.034**	0.073	0.049
Tau PET	Lasso	0.002	0.002	138.95	0.890	0.063	0.120	0.080
	SSL	0.500	10.000	**106.84**	**0.942**	**0.051**	**0.100**	**0.067**
	SSL-IAR	0.3700	8.000	119.73	0.919	0.055	0.104	0.073
	EN	0.002	0.002	134.83	0.903	0.061	0.121	0.077
	SSEN	0.270	10.000	118.36	0.919	0.054	0.114	0.070
	SSEN-IAR	0.270	9.500	118.15	0.924	0.054	0.108	0.070

**Table 2:** ADNI: Classification performance.

	Model	*s*_0_	*s*_1_	Cross-Validated Average						
				AC	SN	SP	PPV	NPV	MCC	F1
Cortical Thickness	Lasso	0.002	0.002	0.937	0.669	0.982	0.864	0.947	0.726	0.753
	SSL	0.270	7.500	0.950	0.751	0.983	0.883	**0.960**	0.786	0.811
	SSL-IAR	0.260	6.000	**0.951**	**0.756**	0.984	0.886	**0.960**	**0.791**	**0.816**
	EN	0.001	0.001	0.943	0.715	0.981	0.865	0.954	0.755	0.783
	SSEN	0.260	10.00	0.949	0.731	0.985	0.891	0.956	0.778	0.802
	SSEN-IAR	0.280	10.00	**0.951**	0.736	**0.987**	**0.903**	0.957	0.788	0.811
Tau PET	Lasso	0.002	0.002	0.920	0.549	0.978	0.796	0.932	0.619	0.649
	SSL	0.500	10.00	**0.933**	**0.617**	0.983	0.850	**0.942**	**0.689**	**0.715**
	SSL-IAR	0.370	8.000	0.927	0.590	0.980	0.827	0.938	0.661	0.689
	EN	0.002	0.002	0.923	0.541	0.983	0.831	0.932	0.632	0.656
	SSEN	0.270	10.00	0.930	0.561	**0.988**	**0.879**	0.935	0.668	0.685
	SSEN-IAR	0.270	9.500	0.930	0.598	0.983	0.845	0.940	0.675	0.700

For these particular data sets we note that all models exhibited relatively good performance in some respects, i.e., AUC near or above 0.90 before classification, and with accuracy, specificity, and NPV above 0.90 after classification. However, the samples in both data sets were unbalanced, with ≈85% of subjects in both data sets being cognitively normal. When we examine metrics that are more sensitive to this imbalance, classification performance drops, and these may be the more important metrics to consider. Specifically, sensitivity is never above 0.76 or 0.62 and MCC is never above 0.80 or 0.70 for the cortical thickness and tau PET data sets, respectively. We therefore draw more attention to these metrics, which for the cortical thickness data are highest for SSL-IAR, and for the tau PET data are highest for SSL.

### Simulation study

**Fig 4:**
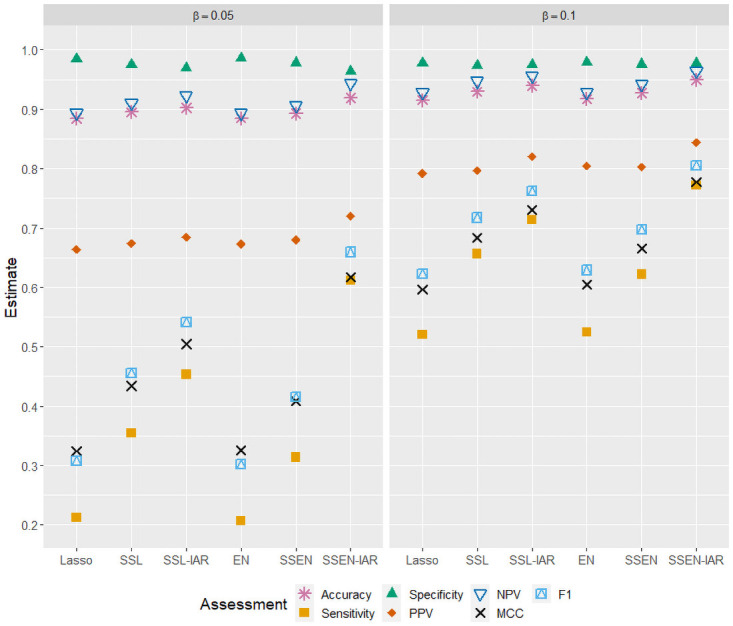
Simulation study classification performance when non-zero parameters are equal to 0.05 (LEFT) or 0.10 (RIGHT).

Table 3 displays deviance, mean squared error (MSE), mean absolute error (MAE), area under the ROC curve (AUC), and misclassification (MC) for each of the six modeling approaches described above. Table 3 displays average prediction errors for each model over the 2,500 simulations. For the larger effect size (non-zero *β*_*j*_ = 0.10), all models perform reasonably well (AUC >0.90), but the spike-and-slab elastic net with IAR priors is best performing model across all metrics with the highest AUC and lowest MSE, MAE, and MC. Note the average model deviance for SSEN-IAR is also much lower than the competing models. As expected, the smaller effect size (non-zero *β*_*j*_ = 0.05) results in decreased model performance for all models, but we see that again SSEN-IAR is the best fitting model across all metrics and in particular is the only model with AUC >0.90 (see Table 3). Note also that the traditional models (lasso and EN) did not outperform their spike-and-slab counterparts on any metric included in Table 3. Table 4 and Fig 4 display average metrics of classification performance over the 2,500 simulations. SSEN-IAR is again the best performing model across all metrics for both parameter sizes. Accuracy, specificity, and NPV are all near or above 0.90 for all models at both parameter sizes. However, the simulations were generated such that in the vast majority of cases the percentage of “events” in a data set was no greater than 20%, which means that accuracy, specificity, and NPV may mislead us regarding model performance. Model differences in sensitivity, MCC, and F1 score show greater disparity across models, where we see that models that include IAR priors on logit probabilities of inclusion, and specifically SSEN-IAR, significantly outperform the other models. At the larger parameter size (non-zero *β*_*j*_ = 0.10), SSEN-IAR and SSL-IAR have sensitivities of 0.7717 and 0.7141, respectively, while other models range from 0.5203 to 0.6564. Similarly, with respect to MCC, SSEN-IAR and SSL-IAR have values of 0.7774 and 0.7305, with other models ranging from 0.5963 to 0.6831. SSEN-IAR is also the only model to achieve an F1 score ≥0.80. Similar patterns are observed at the smaller parameter size (non-zero *β*_*j*_ = 0.05), but again performance decreases compared to the larger parameter size. SSEN-IAR has a sensitivity = 0.611 (other models range from 0.2119 to 0.4532), MCC = 0.617 (other models range from 0.324 to 0.505), and F1 score = 0.659 (other models ranging from 0.307 to 0.541). While SSEN-IAR was the best performing model with respect to PPV at both parameter sizes, model performance was more similar across models (non-zero *β*_*j*_ = 0.10 and *β*_*j*_ = 0.05 ranged from 0.792 to 0.844 and 0.664 to 0.720, respectively). Note also that the traditional models (lasso and EN) did not outperform their spike-and-slab counterparts on any metric except specificity, where all metrics were tightly clustered around 0.97-0.98.

**Table 3:** Simulation study: Average prediction error estimates.

	Model	*s*_0_	Cross-Validated Average					
			*s*_1_	Dev.	AUC	MSE	MAE	MC
*β*_*j*_ = 0.10	Lasso	0.022	0.022	99.91	0.940	0.061	0.126	0.086
	SSL	0.080	1.000	84.14	0.957	0.051	0.103	0.070
	SSL-IAR	0.100	1.000	74.333	0.967	0.045	0.089	0.061
	EN	0.037	0.037	97.78	0.943	0.060	0.124	0.083
	SSEN	0.070	1.000	87.16	0.954	0.053	0.110	0.073
	SSEN-IAR	0.100	2.000	**63.47**	**0.976**	**0.038**	**0.072**	**0.051**
*β*_*j*_ = 0.05	Lasso	0.035	0.035	142.11	0.847	0.086	0.172	0.116
	SSL	0.060	1.000	129.33	0.876	0.078	0.154	0.105
	SSL-IAR	0.090	1.000	119.92	0.898	0.072	0.138	0.097
	EN	0.061	0.061	140.91	0.851	0.085	0.172	0.115
	SSEN	0.050	1.000	132.600	0.868	0.080	0.160	0.107
	SSEN-IAR	0.100	2.000	**102.24**	**0.937**	**0.060**	**0.104**	**0.081**

**Table 4:** Simulation study: Average classification performance.

	Model	*s*_0_	Cross-Validated Average							
			*s*_1_	AC	SN	SP	PPV	NPV	MCC	F1
*β*_*j*_ = 0.10	Lasso	0022	0.022	0.916	0.520	0.978	0.792	0.928	0.596	0.622
	SSL	0080	1.000	0.930	0.656	0.973	0.797	0.947	0.683	0.717
	SSL-IAR	0100	1.000	0.939	0.714	0.975	0.821	0.956	0.731	0.762
	EN	0037	0.037	0.917	0.525	0.979	0.805	0.929	0.605	0.629
	SSEN	0070	1.000	0.927	0.622	0.975	0.803	0.942	0.666	0.697
	SSEN-IAR	0100	2.000	**0.949**	**0.772**	**0.977**	**0.844**	**0.964**	**0.777**	**0.805**
*β*_*j*_ = 0.05	Lasso	0035	0.035	0.884	0.212	0.984	0.664	0.894	0.324	0.307
	SSL	0060	1.000	0.895	0.355	0.975	0.674	0.911	0.434	0.456
	SSL-IAR	0090	1.000	0.903	0.453	0.969	0.685	0.923	0.505	0.541
	EN	0061	0.061	0.885	0.207	0.985	0.673	0.893	0.325	0.302
	SSEN	0050	1.000	0.893	0.314	0.978	0.680	0.906	0.408	0.415
	SSEN-IAR	0100	2.000	**0.919**	**0.611**	**0.964**	**0.720**	**0.944**	**0.617**	**0.659**

## Discussion

In this work we have applied the spike-and-slab elastic net as a classifier, and demonstrated its utility using an analysis of subjects from the ADNI study, where we classified subjects who are cognitively normal or have dementia using several imaging modalities from ADNI data. We also used simulation study to demonstrate that including spatial information in the variable selection process for higher resolution images can further improve classification performance. While this is not the first paper to apply the elastic net to neuroimaging data related to AD, it is to our knowledge the first attempt to apply the spike-and-slab prior framework to classification in AD data; an additional novelty is the explicit modeling of spatial information within the spike-and-slab elastic net framework, which we initially developed and explored in other work [24].

The classification accuracy estimates obtained using ADNI data are often comparable to that of other classification methods in the literature, which suggests that the presented algorithms may prove useful in wider contexts [12]. The models using spike-and-slab priors tended to outperform traditional models with respect to accuracy, specificity, PPV, and NPV, but the difference across models within a classification scenario was often relatively small. The clearest benefit of the spike-and-slab elastic net framework was with respect to sensitivity and MCC, which given the imbalance in the data are arguably the more important metrics to consider. Nevertheless, the traditional models never outperformed their spike-and-slab counterparts, demonstrating that the spike-and-slab framework may have broader potential as a tool for using medical images for classification.

There are limitations related to the data used in the current study, several of which point towards ways in which model performance may be improved. Many classification approaches use atlases to reduce the dimension of the predictors/features [12]. However, given the ability of the presented class of models to handle high dimensional spatial data, restricting analyses to data averaged within each region of the Desikan-Killiany atlas, which reduces thousands of measurements to 68 per subject, may reduce the effectiveness of the models used in this work. While dimension reduction is necessary to fit many models in the first place, variants of the elastic net can handle situations where there are many more predictors/features than subjects, and the addition of the IAR prior on logit inclusion probabilities can also incorporate spatial information into variable/feature selection. Another concern with atlas use is that important and relevant neural effects may follow spatial patterns that do not conform to the atlas used. In this case, effects would not be detectable after reduction to the atlas, harming model performance.

We have addressed these limitations in part by examining the classification performance using simulated data with finer resolution (1600 vs. 68 predictors) and similar outcome imbalance to the ADNI data. In the simulated data the spike-and-slab elastic net with IAR priors was uniformly the best performing model in all scenarios, and especially noticeable compared to its competitors with respect to metrics that better account for imbalance in outcome distribution, e.g, MCC, sensitivity, and F1 score. These results suggest that for finer resolution images we may expect spike-and-slab models with IAR priors on logit inclusion probabilities to significantly outperform both traditional elastic net models and spike-and-slab elastic net models without spatially structured priors, and that the model may be broadly useful for classification of clinical outcomes using medical images in contexts other than AD.

Despite our intuitions regarding dimension reduction, algorithms that use reduced features tend to perform better than those based on voxel or vertex level data [41]. However, even if vertex or voxel level data is too noisy to improve performance, there are a wide range of dimension reduction methods available, including multi-modal approaches to atlas creation [42]. It is also possible that a finer resolution dimension reduction would improve model performance, and take greater advantage of these methods’ strengths. Given that the spike-and-slab elastic net framework is specifically designed to handle “noisy” situations, we may reasonably expect the difference in performance between the spike-and-slab and traditional elastic net to be larger as the number of variables/features increases as the effect sizes of respective regions decreases, which was the case in our simulation study. More flexible approaches to dimension reduction may also yield different, and potentially more relevant, feature sets for tau PET imaging and cortical thickness data sets, which may also improve model performance. We also restricted analysis to cross sectional data, but it would useful to perform a study to determine whether the algorithm could predict whether subjects would progress from cognitively normal to dementia using longitudinal data. Longitudinal prediction is almost certainly a more difficult classification problem, and our simulation results, e.g., the better performance of SSEN-IAR for smaller effect sizes, suggest that the spike-and-slab framework may be a better candidate for longitudinal prediction than the traditional elastic net models.

While there are limitations to the current study, the spike-and-slab elastic net models tended to outperform the traditional elastic net models across several metrics, noticeably improved sensitivity and MCC estimates, and showed comparable classification accuracy to other algorithms in the literature. A simulation study further demonstrated scenarios where spike-and-slab models with spatially structured priors could markedly improve classification performance. In addition, there are many future directions for extending the model, including more flexible dimension reduction as a step before applying the spike-and-slab elastic net, which may allow one to create a feature set that better exploits the strengths of the model, or longitudinal prediction of disease development.
